# Cancer immunohistogram representing cancer-immunity cycle by immunohistochemistry predicts the efficacy of immune checkpoint inhibitors in urological cancer patients

**DOI:** 10.1038/s41598-022-14700-1

**Published:** 2022-06-23

**Authors:** Toshiki Kijima, Terufumi Kubo, Daisaku Nishihara, Akinori Nukui, Yoshihiko Hirohashi, Toshihiko Torigoe, Takao Kamai

**Affiliations:** 1 Department of Urology, Dokkyo Medical University, 880 Kitakobayashi, Mibu, Shimotsuga, Tokyo, 321-0293 Japan; 2 Department of Pathology, Sapporo Medical University, Sapporo, Japan; 3 Department of Urology, Tochigi Cancer Center, Tochigi, Japan

**Keywords:** Urological cancer, Cancer immunotherapy

## Abstract

We developed an immunohistogram representing an individual cancer-immunity cycle based on immunohistochemical analyses. We evaluated its ability to predict the efficacy of immune checkpoint inhibitors (ICI) in 11 patients with urothelial carcinoma and 7 patients with renal cell carcinoma who underwent surgery and received ICIs for disease recurrence. Immunohistochemical analyses for CD8, TIA-1, HLA class I, HLA-DR, and PD-L1 were performed and scored 0–3. T-cell infiltration pattern was classified into desert, excluded, partially inflamed, and inflamed. Tumors with an inflamed or partially inflamed pattern and positive scores (score ≥ 1) for all five immune markers were classified as “immune-hot” and others as “immune-cold.” Association between the immunohistogram and ICI treatment efficacy was evaluated with objective response rate, disease control rate (DCR), progression-free survival (PFS), and cancer-specific survival (CSS). Eight (44%) and 10 (56%) patients had immune-hot and immune-cold tumors, respectively. Immune-hot tumors showed a higher DCR (100% vs. 40%, *p* < 0.01), longer PFS (median unreached for hot, 1.3 months for cold, *p* < 0.01), and longer CSS (median unreached for hot, 3.3 months for cold, *p* < 0.01) than immune-cold tumors. The immunohistogram could be clinically useful as an accessible biomarker for precision cancer immunotherapy in urological cancer.

## Introduction

The recent development of immune checkpoint inhibitors (ICIs) has substantially changed the treatment of urological cancers, including urothelial carcinoma (UC) and renal cell carcinoma (RCC). ICIs are currently approved as a second-line therapy^1^ and first-line switch maintenance therapy^2^ for patients with UC. Several combinations of ICIs with molecular targeted agents have been approved as first-line therapy for RCC^3–7^. Although ICIs can provide robust and durable responses in a subset of patients, their response rates remain unsatisfactory. Therefore, it is essential to identify and develop biomarkers for predicting the efficacy of ICIs.

Several potential biomarkers, such as the expression of programmed death-ligand 1 (PD-L1)^8^, tumor mutation burden^9^, microsatellite instability^10^, somatic copy number alterations^11^, immune inflamed phenotype^12^, T cell repertoire clonality change^13^, and human leukocyte antigen (HLA) class I diversity^14^, have been reported to be associated with response to ICIs across various cancer types. However, none of these factors, as a single biomarker, could sufficiently discriminate individual patients who will respond to ICIs from those who require other therapies. In contrast to conventional anticancer therapies that directly target cancer cells, ICIs affect the immune systems of patients or the tumor microenvironment (TME) to exert anticancer activity. Owing to the complex interaction between tumor cells and TME, a comprehensive understanding of multiple functional pathways that affect tumor-immune interactions is crucial for developing biomarkers for predicting the efficacy of ICIs. To visualize the immunological status of individual patients, Blank et al.^15^ proposed the concept of a cancer immunogram that integrates multiple tumor- and host-specific parameters. Cancer immunograms are useful for understanding complex tumor-immune interactions, indicating compromised steps that prevent ICI efficacy, and proposing possible combination therapies to restore the efficacy of ICI therapy in each patient. Several previous studies^16–18^ have applied similar cancer immunograms to various cancer types including UC^19^. However, immunograms have not been used in clinical practice because they are developed based on RNA-sequencing which lacks clinically well-validated strict standard.

To use the concept of the cancer immunogram in clinical practice for patients with urological cancer, we comprehensively investigated the expression of checkpoint molecules and the status of infiltrating immune cells using immunohistochemical analysis of resected primary tumors. Using these data, we developed an “immunohistogram” to visualize tumor-immune interactions in each patient. The components of the immunohistogram were determined based on the concept of the cancer immunity cycle, which was proposed by Chen et al.^20^ as a dynamic and sequential process to achieve a successful anti-tumor immune response. The patients were classified into immune-hot and immune-cold tumor groups based on the immunohistochemical findings. The association between the immunohistogram and the treatment efficacy of ICIs was evaluated with objective response rate (ORR), disease control rate (DCR), progression-free survival (PFS), and cancer-specific survival (CSS).

## Results

### Patient characteristics

This study involved 18 patients with urological cancer who underwent surgical resection of the primary tumor and received ICIs for residual or recurrent disease thereafter (Table 1). The median age of the patients was 71 (range 41–84) years, and 17 (94%) patients were men. Of these, seven had kidney cancer (six with clear cell RCC, and one with mucinous tubular and spindle cell RCC), five had UC in the upper urinary tract, and the remaining six had UC in the bladder. The median interval between surgery for the primary tumor and ICI therapy was 6 (range 1–246) months, and seven patients had an interval greater than 12 months. Metastatic sites during ICI therapy were the lungs in 8 (44%), local recurrence in 6 (33%), the liver in 4 (22%), and bone in 3 (17%). Among the seven patients with RCC, four received ipilimumab plus nivolumab as the first-line therapy, and the remaining three received nivolumab as the second-line therapy or later setting. All patients with UC relapsed after at least one platinum-based chemotherapy and received pembrolizumab as the second-line therapy.

**Table 1:** Patient’s characteristics.

Patient	Age (y)	Sex	Primary site	Histology of primary tumor	Pathological stage	Interval from surgery to ICI (m)	Metastatic sites at ICI therapy	Administered ICI	Treatment lines of ICI
RCC01	64	M	Kidney	Clear cell RCC	pT2aNx	1	Lung, Liver, LNs	Nivolumab	2nd
RCC02	56	M	Kidney	Clear cell RCC	pT3aN0	1	Lung	Ipilimumab/Nivolumab	1st
RCC03	61	M	Kidney	Clear cell RCC	pT3aNx	1	Lung, Thyroid	Nivolumab	4th
RCC04	84	M	Kidney	Clear cell RCC	pT1bN0	22	Local	Ipilimumab/Nivolumab	1st
RCC05	72	M	Kidney	Clear cell RCC	pT2aN0	4	Lung, Adrenal gland, Local	Ipilimumab/Nivolumab	1st
RCC06	77	M	Kidney	Clear cell RCC	pT3aNx	6	Lung, Adrenal gland	Nivolumab	3rd
RCC07	69	M	Kidney	Mucinous tubular spindle cell RCC	pT2aNx	1	Lung, Bone	Ipilimumab/Nivolumab	1st
UTUC01	61	F	Renal pelvis	UC	pT3N1	69	Bone, Bladder, LNs	Pembrolizumab	2nd
UTUC02	71	M	Renal pelvis	UC	pT3N2	13	Bladder, LNs	Pembrolizumab	2nd
UTUC03	70	M	Renal pelvis	UC	pT3N0	43	LNs	Pembrolizumab	2nd
UTUC04	72	M	Renal pelvis	UC	pT4N0	1	Liver, LNs	Pembrolizumab	2nd
UTUC05	84	M	Ureter	UC	pT3Nx	1	Liver	Pembrolizumab	2nd
BUC01	77	M	Bladder	UC	pT3bN0	6	Lung, Local	Pembrolizumab	2nd
BUC02	82	M	Bladder	UC	pT2N0	1	Local	Pembrolizumab	2nd
BUC03	75	M	Bladder	UC	pT3aN0	12	LNs	Pembrolizumab	2nd
BUC04	82	M	Bladder	UC	pT3bN0	246	Local, LNs	Pembrolizumab	2nd
BUC05	41	M	Bladder	UC	pT2N0	13	Lung, Liver, Bone, Brain	Pembrolizumab	5th
BUC06	71	M	Bladder	UC	pT4aN1	2	Local	Pembrolizumab	2nd

RCC, renal cell carcinoma; UTUC, upper tract urothelial carcinoma; BUC, bladder urothelial carcinoma; ICI, immune checkpoint inhibitor, m, months; LNs, lymph nodes.

### Development of immunohistogram

The results of the immunohistochemical analysis, objective responses to ICI therapy, and survival outcomes after ICI therapy are presented in Table 2. Eight (44%) patients had positive HLA-DR, suggesting functional priming and activation (step 3 in the cancer immunity cycle). Intriguingly, all these patients with positive HLA-DR also had infiltration (inflamed or partially inflamed) of CD8-positive and TIA-1-positive T lymphocytes and positive HLA-class I expression, which led to classifying them under the immune-hot group. In contrast, some patients with negative HLA-DR showed excluded or desert patterns of T-cell infiltration, and a small number of CD8-positive, TIA-1-positive T lymphocytes, causing them to be classified under the immune-cold group. There was a positive correlation between the number of CD8-positive and TIA-1-positive cells, suggesting that most CD8-positive cells evaluated in this study were activated, not exhausted, T cells. Based on the results of immunohistochemical analyses, eight (44%) patients were classified into the immune-hot group and the remaining 10 were classified into the immune-cold group.

**Table 2:** Results of immunohistochemical analysis and treatment outcomes of ICI therapy.

Patient	HLA-DR		CD8		TIA-1		T cell infiltration pattern		HLA class I		PD-L1		FoxP3		Immunohistogram	Response to ICI	PFS	CSS
	Positive area (%)	Score	Cells/HPF	Score	Cells/HPF	Score		Score	Intensity :positive area (%)	Score	Positive area (%)	Score	Cells/HPF	Score			Months	Months
RCC01	60	3	288	3	112	3	Inflamed	3	3:100	3	60	3	5	1	Immune-hot	CR	18.8	18.8
RCC02	50	3	102	3	70	3	Inflamed	3	3:80, 1:20	3	60	3	5	1	Immune-hot	PR	36.5	36.5
RCC03	10	0	50	3	14	2	Excluded	1	2:60, 1:40	2	0	0	0	0	Immune-cold	PR	23.0	52.0
RCC04	50	3	88	3	65	3	Partially inflamed	2	3:50, 1:50	3	30	2	0	0	Immune-hot	CR	30.1	30.1
RCC05	50	3	324	3	172	3	Partially inflamed	2	3:100	3	5	1	15	2	Immune-hot	SD	20.0	28.6
RCC06	80	3	138	3	160	3	Inflamed	3	3:80, 1:20	3	10	1	5	1	Immune-hot	CR	26.2	26.2
RCC07	0	0	9	1	7	1	Excluded	1	3:20, 1:80	1	100	3	0	0	Immune-cold	PR	25.4	25.4
UTUC01	0	0	8	1	3	1	Desert	0	2:30, 1:70	1	0	0	5	0	Immune-cold	PD	1.3	1.3
UTUC02	50	3	114	3	56	3	Partially inflamed	2	3:70, 2:30	3	60	3	10	1	Immune-hot	PR	17.9	17.9
UTUC03	0	0	28	2	13	2	Inflamed	3	3:70, 2:30	3	0	0	0	0	Immune-cold	PD	1.9	13.9
UTUC04	5	0	16	2	2	1	Desert	0	3:20, 0:80	0	5	1	5	1	Immune-cold	PD	0.5	1.6
UTUC05	10	0	29	2	11	2	Partially inflamed	2	3:80, 2:20	3	5	1	5	1	Immune-cold	PD	0.1	1.4
BUC01	0	0	81	3	54	3	Excluded	1	3:100	3	5	1	20	2	Immune-cold	PR	15.4	15.4
BUC02	20	1	82	3	31	3	Partially inflamed	2	3:50, 1:50	3	10	1	5	1	Immune-hot	SD	12.7	17.1
BUC03	0	0	32	3	13	2	Partially inflamed	2	3:80, 1:20	3	5	1	5	1	Immune-cold	SD	11.2	22.2
BUC04	70	3	122	3	63	3	Inflamed	3	3:80, 1:20	3	60	3	20	2	Immune-hot	CR	43.8	43.8
BUC05	0	0	8	1	5	1	Desert	0	3:30, 0:70	0	0	0	0	0	Immune-cold	PD	1.3	3.3
BUC06	0	0	40	3	18	2	Excluded	1	2:20, 0:80	0	30	2	0	0	Immune-cold	PD	1.2	2.8

RCC, renal cell carcinoma; UTUC, upper tract urothelial carcinoma; BUC, bladder urothelial carcinoma; ICI, immune checkpoint inhibitor, PFS, progression free survival; CSS, cancer specific survival.

### Association between immunohistogram groups and objective responses

**Figure 1:**
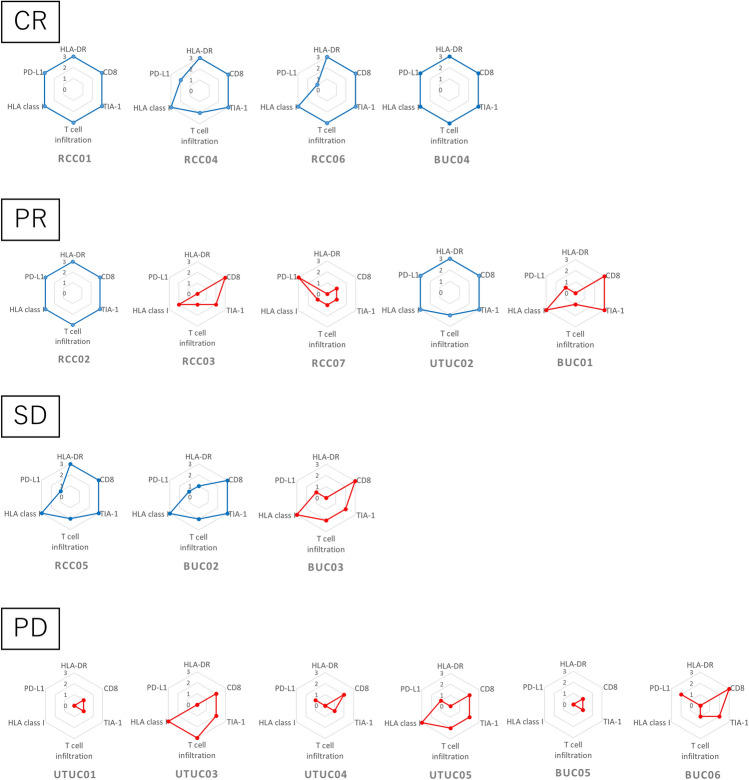
Immunohistograms of all patients stratified according to objective response to ICI therapy. Immunohistograms of the immune-hot group are drawn in blue lines and those of the immune-cold group are drawn in red lines. CR: complete response, PR: partial response, SD: stable disease, PD: progressive disease.

The objective response to the ICI therapy was CR in 4 patients, PR in 5 patients, SD in 3 patients, and progressive disease (PD) in 6 patients. Immunohistograms of all patients were developed based on the immunohistochemical analyses and sorted according to objective responses in Fig. 1. Although both immune-hot and immune-cold groups included patients with PR and SD, patients with CR were observed only in the immune-hot group, and those with PD were observed only in the immune-cold group, suggesting the better response in patients in the immune-hot group. The association between the immunohistogram groups and objective response is summarized in Table 3. The ORR was 75% in the immune-hot group compared with 30% in the immune-cold group, although there was no significant difference between these values. DCR was significantly higher in the immune-hot group than in the immune-cold group (100% vs. 40%, *p* = 0.01).

**Table 3:** Association between immunohistogram and objective response to ICI therapy.

Immunohistogram	CR	PR	SD	PD	ORR (CR + PR) (%)	*p*	DCR (CR + PR + SD) (%)	*p*
Immune-Hot (n = 8)	4	2	2	0	75	0.15	100	0.01
Immune-Cold (n = 10)	0	3	1	6	30		40	

CR, complete response; PR, partial response; SD, stable disease; PD, progressive disease; ORR, objective response rate; DCR, disease control rate.

Three patients showed PR despite being classified into the immune-cold group (RCC03, RCC07, and BUC01). These three patients had negative HLA-DR and excluded patterns in T-cell infiltration. In contrast, there were no patients who did not respond to ICI therapy in the immune-hot group.

Among the seven patients in whom the interval between surgery for primary tumor and ICI therapy was more than 1 year, of the three patients in the immune-hot group (RCC04, UTUC02, and BUC04), 2 showed CR and 1 showed PR, and of the four patients in the immune-cold group (UTUC01, UTUC03, BUC03, and BUC05), 1 showed SD and 3 showed PD. This suggests that the immunohistogram groups are predictive of response to ICI therapy even in patients with a longer interval between primary surgery and ICI therapy.

### Association between the immunohistogram groups and survival outcomes

**Figure 2:**
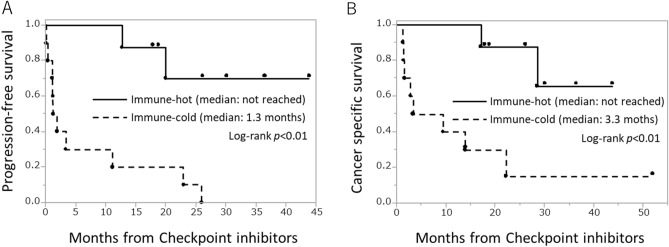
Survival outcomes after ICI therapy according to immunohistogram groups. (**A**) Progression-free survival (PFS), (**B**) cancer-specific survival (CSS).

The median follow-up time in the immune-hot group, immune-cold group, and all patients was 17.5, 8.4, and 14.7 months, respectively. Patients in the immune-hot group had significantly longer PFS (median unreached vs 1.3 months, *p* < 0.01) and longer CSS (median unreached vs 3.3 months, *p* < 0.01) than those in the immune-cold group (Fig. 2). According to Lakatos’s method^21^, the required sample size to detect the differences in PFS is 8 (4 in each group) with statistical power of 0.854, and the required sample size to detect the differences in CSS is 10 (5 in each group) with statistical power of 0.847. The immune-cold group was an independent predictor of worse CSS (HR 14.2, 95% CI 1.84–408, *p* = 0.01) according to the Cox proportional hazard model (Table 4).

**Table 4:** Cox proportional hazard model for cancer specific survival after ICI therapy.

Variable	Category	HR	95% CI	*p*
Pathological T stage	T3-4	1.01	0.18–7.6	0.99
	T1-2 (ref)	1		
Grade	G3	3.26	0.36–101	0.32
	G2 (ref)	1		
Immunohistogram	Immune-Cold	14.2	1.84–408	0.01
	Immune-Hot (ref)	1		

HR, hazard ratio; CI, confidence interval.

## Discussion

In the present study, we developed an immunohistogram based on immunohistochemical analysis of resected primary cancer tissues as a potential clinically available biomarker for ICI therapy in patients with urological cancer. Immunohistogram describes the status of the following steps of cancer immunity cycle: step 3, priming and activation (HLA-DR); step 4, trafficking of T cells to tumors; step 5, infiltration of T cells into tumors (CD8 and TIA-1); step 6, recognition of cancer cells by T cells (HLA class I); and step 7, killing of cancer cells (PD-L1). Patients with tumors that showed positive scores for all the above factors were assumed to have functional cancer immunity cycle; therefore, we classified them as immune-hot group. We confirmed that the immune-hot group, which suggests the existence of a functional cancer immunity cycle, was significantly associated with a higher DCR, longer PFS, and CSS after ICI therapy. Some patients were classified into the immune-cold group and did not respond to ICI therapy even though their tumors showed infiltration of CD8-positive T cells, suggesting that the evaluation of the entire cancer immunity cycle, not a single biomarker, is important in the prediction of ICI efficacy. The immunohistogram helps visualize the status of each step in the cancer-immunity cycle and therefore, it is useful for not only predicting the efficacy of ICI therapy but also discussing treatment targets to improve ICI efficacy. To our knowledge, this is the first study to evaluate the utility of an immunohistogram as a predictive biomarker for ICI therapy.

The feasibility of immunohistochemical analyses in pathological diagnostic workflows has prompted extensive efforts to establish the utility of immunohistochemical analyses of PD-L1 as a potential biomarker for ICI therapy^22^. In pivotal clinical trials of ICI therapy for urological cancer, the predictive ability of PD-L1 and related biomarkers has been studied using IHC. In RCC trials, which evaluated ICIs in combination with conventional molecular-targeted agents, high PD-L1 expression was associated with a treatment benefit of ICIs^3–5^. In UC trials, the survival benefit of avelumab maintenance therapy was observed in the PD-L1-high population in the JAVELIN Bladder 100 studies^2^, whereas the second line pembrolizumab study (Keynote-045) reported a similar response irrespective of PD-L1 combined positive score^1^. As reported by the above-mentioned studies, PD-L1 classification combining the expression on tumor and immune cells might be useful for predicting ICI efficacy. However, owing to differences in the antibodies used, staining methods, and scoring algorithms, there is a major concern about the interchangeability and comparability of data for clinical use^23^. Furthermore, PD-L1 classification might combine predictive and prognostic information, which may have led to inconsistencies in the ability of PD-L1 expression to predict outcomes of ICI therapy in previous trials.

The importance of tumor-infiltrating lymphocytes (TILs) in cancer immunity has been demonstrated by numerous oncological studies. TILs include various functionally divergent components, such as active or exhausted CD8-positive T cells, regulatory T cells, and natural killer (NK) cells. In RCC, it has been reported that an increased number of CD8-positive T cells in the TME is associated with a better prognosis^24^, whereas an increase in exhausted T cells, which also belong to CD8-positive T cells, is associated with shorter survival^25^. Similarly, higher CD8-positive T cells in bladder cancer are associated with better outcomes^26^. However, the predictive ability of TILs for the efficacy of ICI therapy has not yet been established. Based on the expression of PD-L1 and the presence or absence of TILs, Teng et al.^27^ proposed classifying TME into four groups. We applied this classification to patients with surgically treated upper tract urothelial cancer (UTUC) and confirmed its prognostic ability^28^. Although the predictive ability of this classification for the efficacy of ICI therapy has not been well studied, combining multiple factors may improve its predictive ability. In this regard, the concept of immunohistogram, which involves not only TILs and PD-L1 but also the status of HLA molecules, could be useful to develop better biomarkers that are predictive of the efficacy of ICI therapy.

In addition to PD-L1 and TILs, the expression of HLA molecules in the TME is an important factor in predicting the functional cancer immunity cycle. HLA-DR, expressed on antigen-presenting cells such as macrophages, B cells, and dendritic cells, presents cancer antigens to helper T cells to stimulate the immune system. Therefore, we used HLA-DR as a marker for step 3 (priming and activation) in the cancer immunity cycle. In this study, all patients with positive HLA-DR also presented infiltration of CD8-positive and TIA-1-positive T cells and expression of HLA-class I molecules in tumor cells, and they responded to ICI therapy. This suggests that the cancer immunity cycle probably functioned properly in these patients. In contrast, patients with negative HLA-DR tended to show a lower number of CD8-positive, TIA-1-positive T cells, or excluded or desert infiltration patterns, and did not respond to ICI therapy, suggesting an impaired cancer immunity cycle in these patients. The function of HLA-class I is to display peptide fragments of cancer antigens and trigger the attack by cytotoxic T cells. Therefore, the expression of HLA-class I in tumor cells is a prerequisite for cytotoxic T cell-based cancer therapy, and we assume HLA-class I to be a marker for step 6 (recognition of cancer cells by T cells) in the cancer immunity cycle. In this study, the patients with negative HLA-class 1 showed negative HLA-DR and a small number of CD8-positive, TIA-1-positive T cells, suggesting an impaired cancer immunity cycle.

A major concern in the development of an immunohistogram using primary tumor tissue is the possible discrepancy in the TME between the primary tumor and metastatic sites. As tumor cells with high antigenicity are under attack by anti-tumor host immunity, tumor cells with impaired HLA-class I expression can escape from the immune system and become predominant in recurrent or metastatic tumors. We previously referred to this phenomenon as “adaptive immune escape”^29^ and reported this phenomenon in a patient with RCC^30^. Although there were no patients with negative HLA-class I but positive HLA-DR in their primary tumor in this study, evaluation of recurrent or metastatic tumors using IHC should be performed in future trials to confirm the adaptive immune escape phenomenon. On the contrary, we observed a phenomenon opposite to adaptive immune escape in the current study, three patients responded to ICI therapy despite their primary tumor having an immune-cold pattern. As these three patients had negative HLA-DR and the excluded pattern of T-cell infiltration, this immunohistogram pattern might be associated with a discrepancy in the TME between primary tumor and recurrent or metastatic tumors, which warrants further confirmation.

Our study had several limitations. First, this was a retrospective study with a small number of patients; therefore, it was subjected to a probable selection bias and no solid conclusion could be stated. Second, this study is a pan-cancer analysis that included both RCC and UC. A cancer type-specific analysis with an extended cohort is warranted in the future. As the cancer immunity cycle can be applied to all cancer types, pan-cancer analyses might be an optimal method for preliminary analyses, as is the case in previous immunogram studies^18^. Third, we used archival tissue of resected primary cancer to develop cancer immunohistogram; therefore, patients who had undergone tumor biopsy only or those who did not undergo biopsy before ICI therapy were excluded from the study.

In conclusion, immunohistogram, which could be developed only with immunohistochemical analyses, could be associated with DCR and survival of patients with urological cancer treated with ICI therapy. Although a validation study with larger cohort is warranted, immunohistogram could be a clinically accessible potential biomarker for predicting the efficacy of ICI therapy. A more precise and tumor-specific model of the immunohistogram should be investigated in future studies.

## Patients and methods

### Patients

We retrospectively identified 18 patients with urological cancer, including 11 with UC and 7 with RCC, who initially underwent resection of the primary tumor and received ICI therapy for recurrent diseases between January 2018 and April 2020 at Dokkyo Medical University. The study was in accordance with the tenets of the Declaration of Helsinki. The protocol of this retrospective study was approved by the Institutional Review Board of Dokkyo Medical University (approval number: R-31-10J). All patients who were alive at the time of data collection were informed about the study and written informed consent was obtained from them.

### Immunohistochemical analysis and development of immunohistogram

According to the concept of Chen et al.^20^, the cancer immunity cycle consists of the following seven steps: (1) release of cancer antigens; (2) cancer antigen presentation; (3) priming and activation; (4) trafficking of T cells to tumors; (5) infiltration of T cells into tumors; (6) recognition of cancer cells by T cells; and (7) killing of cancer cells. To evaluate each step in the cancer immunity cycle using immunohistochemistry (IHC), the following molecules were selected for immunohistochemical staining: human leukocyte antigen (HLA)-DR representing step 3, CD8 and TIA-1 representing steps 4 and 5, HLA class I representing step 6, and PD-L1 representing step 7. Sections of formalin-fixed, paraffin-embedded tissues of resected primary tumors were immunostained using primary antibodies against CD8 (clone: 4B11, Leica, Wetzlar, Germany), TIA-1 (clone: TIA-1, Abcam, Cambridge, UK), HLA class I (clone: EMR8-5, Hokudo, Sapporo, Japan), HLA-DR (clone: TAL-1B5, Abcam), and PD-L1 (clone: E1L3N, Cell Signaling Technology, Danvers, MA, USA). In addition to the above molecules constituting the immunohistogram, the expression of FoxP3 (clone: 236A/E7, Abcam) was evaluated using IHC, although it is not included in the concept of the cancer immunity cycle. The T-cell infiltration pattern, which represents step 5 of the cancer immunity cycle, was also evaluated using hematoxylin–eosin-stained tissue.

All specimens were reviewed independently by two pathologists (T. Kubo and T. T) who were blinded to the clinical history of the samples, and the results of the immunohistochemical analysis were scored on a scale of 0 to 3. CD8, TIA-1, and FoxP3 were evaluated by the number of positive cells/HPF (high power field) and scored on a scale of 0 to 3, score 0, 0–1; score 1, 2–10; score 2, 11–30; and score 3, > 30. PD-L1 was evaluated by the proportion of positive area as follows: score 0, 0%; score 1, 1%–10%; score 2, 11%–50%; and score 3, > 50%. HLA class I was evaluated by membrane intensity in > 50% of the total area stained and categorized into score 0, none; score 1, weak; score 2, moderate; and score 3, strong. HLA-DR was evaluated by the proportion of positive area as follows: score 0, 0%–10%; score 1, 11%–50%; score 2, > 50% area without positive tumor cells; and score 3, > 50% area with positive tumor cells. T cell infiltration pattern was categorized into score 0, desert; score 1, excluded; score 2, partially inflamed; and score 3, inflamed.

To visualize the functional status of the cancer immunity cycle in each patient, an immunohistogram was drawn as a radar plot with six axes, namely, CD8, TIA-1, HLA class I, HLA-DR, PD-L1, and T-cell infiltration pattern. Tumors with inflamed (score 3) or partially inflamed (score 2) T cell infiltration pattern and positive scores (score ≥ 1) for the remaining five parameters were classified as “immune-hot” tumors and others were classified as “immune-cold” tumors. Constitutively, the immune-hot and immune-cold tumor groups will be referred to as immunohistogram groups.

### Statistical analyses

Chest and abdominal computed tomography (CT) scans were performed at baseline and every 8–12 weeks, or as clinically indicated. Objective response was determined according to the RECIST version 1.1. Clinical benefit was represented by ORR, which is defined as the proportion of patients who show a partial response (PR) or complete response (CR) to therapy and DCR, which is defined as the proportion of patients who have achieved CR, PR, and stable disease (SD) to therapy. PFS was calculated from the start of ICI therapy to the date of progression or death or censored at the last follow-up date. CSS was calculated from the start of ICI therapy to the date of cancer-related death or censored at the last follow-up date. All relevant clinical and pathological data are supplied as supplemental data.

The primary endpoint of this study was to evaluate whether there was any relevant association between the immunohistogram groups and the efficacy of ICI therapy represented by ORR, DCR, PFS, and CSS in patients with urological cancer. The association between the immunohistogram group and ORR or DCR was evaluated using Fisher’s test. PFS and OS were estimated using the Kaplan–Meier method. The association between the immunohistogram groups and PFS or OS was assessed using the log-rank test and Cox proportional hazard model. All statistical analyses were performed using JMP 13.0 (SAS institutes, Cary, NC, USA), and results with *p*-values < 0.05 were considered statistically significant.

## Supplementary Information

Supplementary Information.

## Data Availability

All data generated or analyzed during this study are included in this published article and its supplementary information files.
